# Phylogenetic Variation of *Periophthalmus* Species Living in the Mekong Delta, Vietnam, Using *Cytb* Sequence

**DOI:** 10.1002/ece3.71389

**Published:** 2025-05-07

**Authors:** Quang Minh Dinh, Tran Thi Huyen Lam, Gieo Hoang Phan, Ton Huu Duc Nguyen

**Affiliations:** ^1^ Faculty of Biology Education School of Education, Can Tho University Can Tho Vietnam; ^2^ Center for Management of Laboratories ‐ Practices, Cuu Long University Long Ho District Vinh Long Vietnam; ^3^ Faculty of Agriculture and Rural Development, Kien Giang University Chau Thanh District Kien Giang Vietnam

**Keywords:** *Cytb*, genus *Periophthalmus*, mudflat areas, mudskippers, phylogenetic tree

## Abstract

Mudskippers are amphibious fish that breathe air and have adapted to living on mudflats. They possess unique morphological, physiological, and behavioral features that set them aside from fish that live entirely in water. These characteristics may also have an impact on their phylogenetic and phylogeographical aspects. A study investigated the genetic relationships of mudskippers of the genus *Periophthalmus* found in four mudflat areas in the Mekong Delta. The outcomes of the *Cytb* phylogenetic tree analysis of the genus *Periophthalmus* revealed that the three species of *Periophthalmus*, namely *Periophthalmus chrysospilos* , *Periophthalmus gracilis* , and *Periophthalmus variabilis* , separated into three sub‐clades. The sub‐clades showed a high degree of genetic divergence and were supported by a bootstrap value of 100%. Thus, the *Cytb* gene analysis technique helped to reconstruct the genetic relationship of these three mudskipper species of the genus *Periophthalmus*. This study provided an essential understanding of the evolutionary history of mudskippers and shed light on the phylogenetic and phylogeographical characteristics of this unique group of amphibious fish.

## Graphical abstract

This study investigates the genetic relationships of three Periophthalmus mudskipper species ( *P. chrysospilos* , *P. gracilis* *,* and *P. variabilis* ) from the Mekong Delta using mitochondrial cytochrome b (*Cytb*) gene analysis. The findings reveal significant interspecific genetic divergence (15%–18%) and distinct phylogenetic sub‐clades supported by morphological differences and high bootstrap values. These results provide valuable insights into the evolutionary history and genetic diversity of amphibious fish in this region.

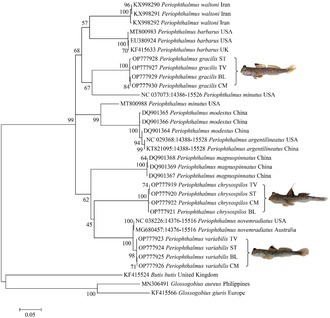

## 1 Introduction

The term “mudskipper” usually refers to the four genera: *Boleophthalmus*, *Periophthalmodon*, *Periophthalmus*, and *Scartelaos* of oxudercine gobies (Oxudercidae) (Ishimatsu and Gonzales 2011). Amphibious fish can withstand long periods of being out of the water, emerging as the tide is on the ebb to feed and find mates (Jaafar and Murdy 2017). Their known habitats are mudflats, creeks, lagoons, and mangroves in the Indo‐Pacific (Baeck et al. 2008). Murdy (1989) noted that among the mudskippers, the *Periophthalmus* genus exhibits the most comprehensive distribution, ranging from the West African coast to the Polynesian archipelago. In Vietnam, Tran et al. (2013) recorded the presence of *Periophthalmus chrysospilos* , *Periophthalmus gracilis* , and *Periophthalmus variabilis* in the Mekong Delta. There are currently no reports of the presence of the three *Periophthalmus* species outside the Mekong Delta region. In the field, these mudskippers are powerful predators with a wide range of prey consisting of crabs, shrimp, small fish, and terrestrial arthropods (Jaafar and Murdy 2017).

The flesh of mudskipper has high nutritional value, so this fish is considered an economically valuable species in many nations (Akinrotimi et al. 2013). Mudskipper flesh contains essential amino acids such as lysine (9.37%), leucine (8.22%), and valine (4.97%), and non‐essential amino acids such as glutamate (16.92%), aspartate (10.71%) and alanine (6.04%) should be good for health (Ridho et al. 2019). In addition, mudskippers are being exploited as bait in hook fishing or used as a traditional medicine to treat frequent urination in children in India (Kanejiya et al. 2017) and as a drug with aphrodisiac properties (Clayton 1993). Many species of mudskippers are traded as ornamental fish (Jaafar and Murdy 2017). Two mudskipper genera—*Periophthalmodon* and *Periophthalmus*—are commonly available to aquarists (Monks 2006).

The taxonomy of members of the family Oxudercidae (formerly known as Gobiidae) is often hampered by morphological similarities, causing considerable difficulties in species identification (Thacker 2003). This has been reported in mudskippers of the genera *Boleophthalmus* and *Periophthalmus*, with many cryptic species reported (Chen et al. 2014; Polgar et al. 2014; Aji and Arisuryanti 2021). Applying traditional morphological methods alone has been shown to be inadequate for accurate identification of *Periophthalmus* species, with some cases of misidentification such as *Periophthalmus walailakae* with *Periophthalmodon schlosseri* (Jaafar et al. 2006), *Periophthalmus takita* with *Periophthalmus novaeguineaensis* Eggert, 1935 (Jaafar and Larson 2008), and *Periophthalmus variabilis* Eggert, 1935 with *Periophthalmus novemradiatus* Hamilton, 1822 (Jaafar et al. 2009). The reason may be that those species have reduced morphology, or in other words, they have lost some morphological characteristics (Agorreta et al. 2013). Therefore, there is a lot of morphological variation within species, making identification difficult. To avoid these limitations, it is crucial to incorporate molecular approaches, such as DNA barcoding, into the taxonomy process. Phylogenetic studies can be performed not only based on morphology but also by molecular approaches. In studying the relationships of lineages within a group of organisms, DNA sequences have been used as a source of traits in phylogenetic studies. DNA sequence data can help clarify Oxudercid relationships without considering the potentially confusing morphological reductions that occur in morphological character analysis (Larson 2012). Bingpeng et al. (2018) noted the main advantages of DNA barcoding over traditional morphological methods. First, it is difficult to distinguish some species with highly similar external morphological features based only on morphology. Second, morphological differences can vary considerably during the different growth periods, yet DNA barcodes can identify individuals at any stage in their development. Third, DNA barcoding techniques can detect cryptic species that share the same morphology but are genetically different and inadvertently classified as the same species.

Ward et al. (2005) and Viswambharan et al. (2015) mentioned the mitochondrial Cytochrome b (*Cytb*) genes are valuable and widely used to identify fish. *Cytb*, a protein‐coding gene in mitochondrial DNA, is used as a molecular marker in low‐level genetic analyses (Arif and Khan 2009). This gene is involved in oxidative phosphorylation (Hatefi 1985). The codons in *Cytb* evolve rapidly and slowly to have some conservative and variable parts (Farias et al. 2001). The *Cytb* gene has many variations and evolves rapidly, making it suitable for identifying variations at the species level or below (Bruford et al. 2003). Therefore, *Cytb* is the DNA barcode used in this study to identify the phylogeny among three species in the *Periophthalmus* genus collected from the estuaries of the Vietnamese Mekong Delta, as well as to determine whether the morphological differences among them are due to morphological plasticity.

## 2 Materials and Methods

### 2.1 Sample Collection

**FIGURE 1:**
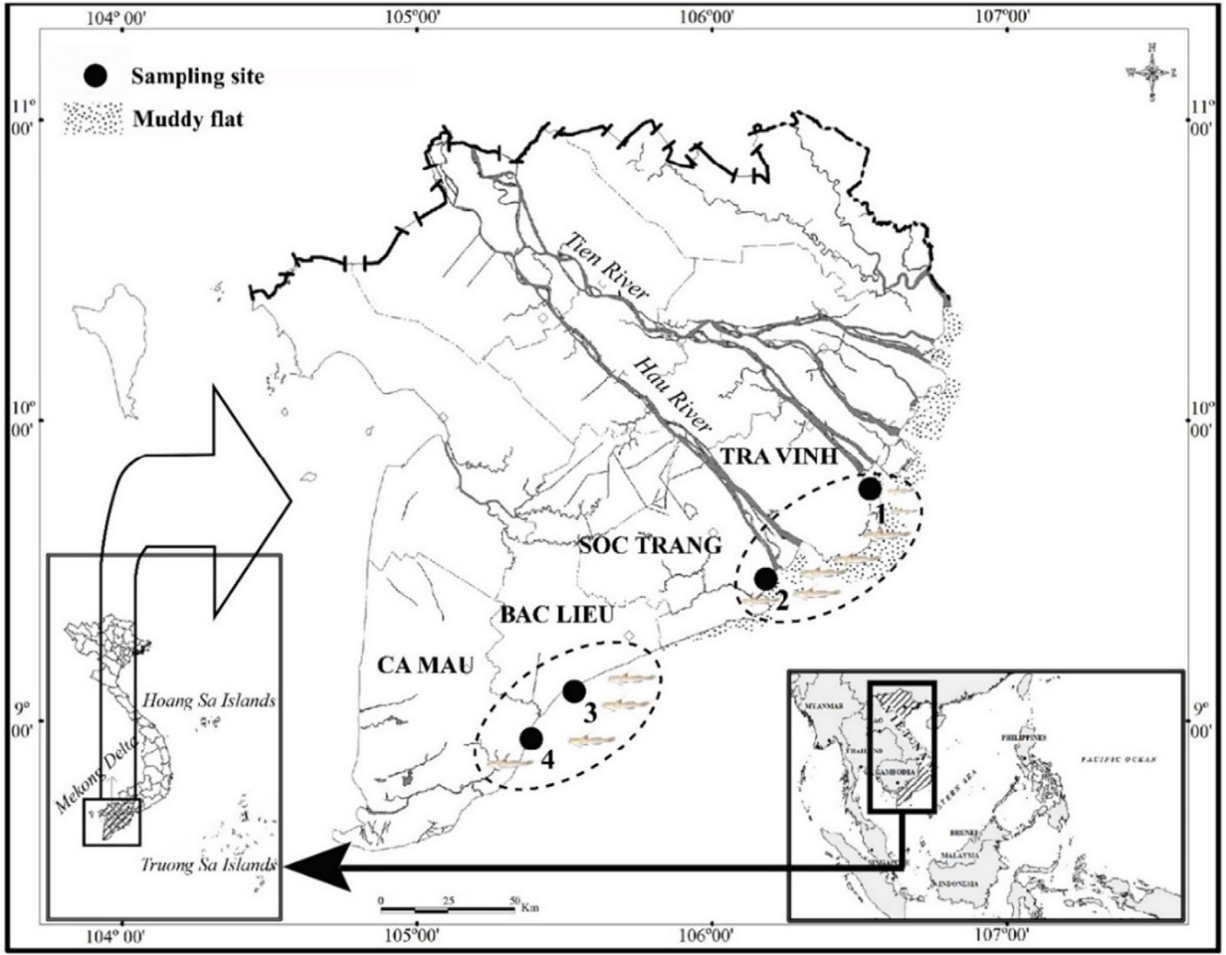
The sampling map in the Mekong Delta, Vietnam (•: Sampling site; 1: Tra Vinh; 2: Soc Trang; 3: Bac Lieu; 4: Ca Mau) (Source: modified from Dinh 2017).

A total of 12 mature samples of *Periophthalmus*, three samples for each species, were collected from four different sites (Table 1) in the Mekong Delta (Figure 1) from April 2020 to March 2021 consisting of Tra Vinh (9°40′29.5″N 106°34′49.5″ E); Soc Trang (9°26′19.7″N 105°10′48.1″ E); Bac Lieu (9°05′50.5″N 105°29′54.7″ E) and Ca Mau (8°58′10.4″N 105°22′58.9″ E). These sites were selected for sampling to represent the natural mudflat areas in the Mekong Delta, which were heavily influenced by the Mekong River flow. Furthermore, these sites were reported to have abundant mudskipper species (Dinh et al. 2022). The specimens were identified based on external morphological characteristics described in Table 2 and are consistent with previous descriptions by Jaafar and Murdy (2017) and Tran et al. (2013).

**TABLE 1:** Coordinates and description of sampling sites.

Sampling sites	Coordinates	Description
Tra Vinh	9°41′18.6″N, 106°30′35.8″ E	Brackish mudflat surrounded by mangroves with the dominant plant of *Sonneratia caseolaris* and *Avicennia marina*
Soc Trang	9°29′26.8″N, 106°11′58.5″ E	Brackish mudflat surrounded by mangroves with the dominant plant of *Sonneratia caseolaris* and *Avicennia marina*
Bac Lieu	9°06′03.2″N, 105°29′49.1″ E	Brackish mudflat surrounded by mangroves with the dominant plant of *Avicennia marna* and *Bruguiera gymnorrhiza* (L.) Savigny
Ca Mau	8°58′17.5″N, 105°22′51.8″ E	Brackish mudflat surrounded by mangroves with the dominant plant of *Avicennia marna* and *Bruguiera gymnorrhiza* (L.) Savigny

**TABLE 2:** The divergence in the morphology of three species in the *Periophthalmus* genus.

Species	Morphological characteristics
*Periophthalmus chrysospilos* Bleeker, 1853 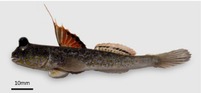	There are many small orange dots on the head and body. The first dorsal is yellow to orange, edged by black and white stripes. Two spines of the first dorsal fin are long filamentous in males. The two fused pelvic fins are disc‐shaped. There is a row of teeth in the upper jaw.
*Periophthalmus variabilis* Eggert, 1935 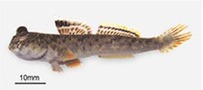	The color of the body is beige or olive brown. The abdomen is milky white with lots of irregular small black spots. The first dorsal is triangular and without filamentous spines. A black stripe in the middle and pale yellow at the root of the second dorsal fin. Pelvic fin rays separate, but spines are conjoined. There is a row of teeth in the upper jaw.
*Periophthalmus gracilis* Eggert, 1935 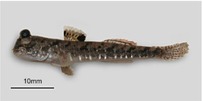	The color of the body is grayish brown with many irregular narrow white horizontal lines. The first dorsal fin is yellowish brown with large black streaks and a white distal margin. Two pelvic fins separate. There is a row of teeth in the upper jaw.

The mudskipper fish was sampled monthly by hand‐catching at the mudflat in each sampling site. The mudskipper samples were then cleaned, and preliminary species were classified based on the outward feature (Table 2). Mudskipper fishes were anesthetized with tricaine methanesulfonate solution (MS222) and kept in absolute cold alcohol (Animal Welfare Assessment No. BQ2020‐03/KSP), then transported to the Lab and reserved at −20°C till use for DNA isolation.

### 2.2 Molecular Procedures

#### 2.2.1 DNA Extraction

Fish genome DNA was isolated from dorsal muscle tissue following the manufacturer's protocol with the TopPURE Genomic DNA Extraction Kit (ABT, Vietnam).

#### 2.2.2 DNA Amplification

The partial *Cytb* mitochondrial gene with sizes ~1300 bp was amplified by the primers GcytbH (5′‐GACTTGAAAAACCACCGTTG‐3′) and GcytbL (5′‐CTCCGATCTCCGGATTACAAGAC‐3′) (Ju et al. 2016). PCR reactions were brought out in 50 μL mixes containing 2 μL of genomic DNA (10–100 ng), 40 μL DEPC‐treated water, 5 μL PCR Buffer 10×, 1.5 μL each primer (10 pmol/μL), and EZ Mix (PhuSa Biochem LTD. Company) consisted of Taq (2 U) and dNTPs (200 μL each dNTP). The negative control sample was the DNA‐reduced water to evaluate the PCR reaction efficiency. The conditions of the PCR reaction were 5 min pre‐denaturation at 95°C; next to 35 cycles: denature at 95°C for 30 s, anneal at 56°C for 30 s, and extend at 72°C for 45 s; finally, extend for 5 min at 72°C and hold at 25°C for 2 min.

#### 2.2.3 Electrophoresis and Sequencing

PCR products were tested on 2% agarose gels stained with SyBr Green I (Lumiprobe—USA) in Tris‐Borate‐EDTA buffer (TBE) at 110 V for 40 min. Electrophoresis gel was then imaged under UV light to check the PCR product band. PCR Purification Kit (Jena Bioscience) was used to purify PCR products according to the manufacturer's directions. All amplification samples were shipped to PhuSa Biochem LTD Co., Can Tho, Vietnam, for forward and reverse bidirectional sequencing using the ABI 3500 sequencer (Thermofisher) according to the manufacturer's instructions.

### 2.3 Dataset Preparation

#### 2.3.1 Sequence Editing

Data obtained from DNA sequences were checked and edited in the Bioedit v.7.2 program (Hall 1999). The chromatograms of each sequence were checked manually to check for ambiguous bases and stop codons. The new sequences have been registered on GenBank with accession numbers OP777919–OP777922, OP777923–OP777926, and OP777927–OP777930 (Table 3). The BLAST tool from NCBI was used to compare the similarity between newly generated *Periophthalmus* sequences with genetic data already available in GenBank (BLAST, http://blast.ncbi.nlm.nih.gov/).

**TABLE 3:** Accession numbers of all *Cytb* gene sequences in this research.

Species	Notes	Locality	Accession numbers registered on NCBI
*Periophthalmus chrysospilos*	Study sample	Tra Vinh	OP777919
		Soc Trang	OP777920
		Bac lieu	OP777921
		Ca Mau	OP777922
*Periophthalmus variabilis*	Study sample	Tra Vinh	OP777923
		Soc Trang	OP777924
		Bac lieu	OP777925
		Ca Mau	OP777926
*Periophthalmus gracilis*	Study sample	Tra Vinh	OP777927
		Soc Trang	OP777928
		Bac lieu	OP777929
		Ca Mau	OP777930
*Periophthalmus waltoni*	Control sample	Iran	KX998290 KX998291 KX998292
*Periophthalmus magnuspinnatus*	Control sample	China	DQ901367 DQ901368 DQ901369
*Periophthalmus modestus*	Control sample	China	DQ901364 DQ901365 DQ901366
*Periophthalmus minutus*	Control sample	USA	MT800988 NC_037073:14386‐15526
*Periophthalmus barbarus*	Control sample	USA USA United Kingdom	EU380924 MT800983 KF415633
*Periophthalmus argentilineatus*	Control sample	USA China	NC_029368:14388‐15528 KT821095:14388‐15528
*Periophthalmus novemradiatus*	Control sample	USA Australia	NC_038226:14376‐15516 MG680457:14376‐15516
*Butis butis*	Control sample	United Kingdom	KF415524
*Glossogobius aureus*	Control sample	Philippines	MN306491
*Glossogobius giuris*	Control sample	Europe	KF415566

#### 2.3.2 Align the Sequences

ClustalW on the MEGA 7.0 program (Kumar et al. 2016) was applied to align the edited *Cytb* of mudskippers.

### 2.4 Genetic Diversity and Phylogenetic Analyses

#### 2.4.1 Nucleotide Composition and Genetic Distance

Bioedit v.7.2 software (Hall 1999) was used to estimate the nucleotide percentage of the *Cytb* sequences. Meanwhile, the MEGA 7.0 program was used to estimate the interspecific genetic distances with the Kimura‐2 Parameter (K2P) model.

#### 2.4.2 Phylogenetic Relationship

The phylogenetic tree was reconstructed using the Maximum Likelihood (ML) method with 1000 bootstraps in MEGA 7.0 software. This method was applied in many studies on phylogenetic relationships in fish by authors such as Agorreta and Rueber (2012), Agorreta et al. (2013), and Çiftci et al. (2013). Merl et al. (2005) emphasized that it is a highly reliable tool for testing the presence of positive selection at the codon level based on sequence evolution in a stochastic model.

## 3 Results and Discussion

### 3.1 PCR Amplification and Species Identification

The amplification of the *Cytb* mitochondrial gene from 12 mudskipper specimens from the mudflat areas in the Mekong Delta produced a fragment of about 1300 bp in length. Sequence lengths after chromatographic‐based editing ranged from 1135 to 1140 bp.

Sequences in GenBank are considered to be most similar when the maximum and total scores are the same, query coverage is close to 100%, and identity is close to 100%. Based on these criteria, all sequences had low similarity to three reference species in GenBank: *P. minutus* , *P. magnuspinnatus* , and *P. barbarus* . As there is not much data on the three mudskippers kept in GenBank, the identity percentage is low.

At a glance at Table 4, all 12 samples of mudskipper in the present study were identified as belonging to only one genus, *Periophthalmus*. The similarity of the samples in the study with the data in GenBank is relatively low, about 86.20%–87.20%. This result suggested that they belong to the same genus, *Periophthalmus*, but are different species. All of the *Cytb* gene sequences of mudskipper samples have been submitted in GenBank with accession numbers OP777919–OP777922 for *P. chrysospilos* , OP777923–OP777926 for *P. variabilis* , and OP777927–OP777930 for *P. gracilis* .

**TABLE 4:** Results of the BLAST searches conducted in the GenBank database using the new sequences of *Periophthalmus* generated in this study as a query.

No.	Fish samples	Reference species from GenBank	Similarity (%)	Query cover (%)	Accession number	References
1	*P. chrysospilos* TV	*P. minutus*	86.26	99	MT800988	(1)
2	*P. chrysospilos* ST	*P. minutus*	86.20	99	MT800988	(1)
3	*P. chrysospilos* BL	*P. minutus*	86.29	99	MT800988	(1)
4	*P. chrysospilos* CM	*P. minutus*	86.34	99	MT800988	(1)
5	*P. variabilis* TV	*P. magnuspinnatus*	86.71	99	DQ901370	(2)
6	*P. variabilis* ST	*P. magnuspinnatus*	86.65	99	DQ901370	(2)
7	*P. variabilis* BL	*P. magnuspinnatus*	86.73	99	DQ901370	(2)
8	*P. variabilis* CM	*P. magnuspinnatus*	86.69	99	DQ901370	(2)
9	*P. gracilis* TV	*P. barbarus*	87.01	99	KF415633	(3)
10	*P. gracilis* ST	*P. barbarus*	86.95	99	KF415633	(3)
11	*P. gracilis* BL	*P. barbarus*	87.18	99	KF415633	(3)
12	*P. gracilis* CM	*P. barbarus*	87.20	99	KF415633	(3)

Abbreviations: (1), Steppan et al. (2022); (2), Wang (2006); (3), Agorreta et al. (2013); BL, Bac Lieu; CM, Ca Mau; ST, Soc Trang; TV, Tra Vinh.

### 3.2 Nucleotide Composition

In general, three mudskipper species had different nucleotide compositions. Specifically, in *P. chrysospilos* , the nucleotide compositions of C were highest, while in *P. gracilis* , %T was highest, and in *P. variabilis* , %C and %T were equivalent (Table 5). All three species had %AT content higher than %GC; however, %AT was highest in *P. gracilis* , followed by *P. variabilis* , and lowest in *P. chrysospilos* . The variation in nucleotide percentage indicated variation between the three *Periophthalmus* species.

**TABLE 5:** The variation in nucleotide percentage (%) of the *Cytb* gene sequence.

Species	Sampling sites	Number accession	Length (bp)	%A	%C	%G	%T	%AT	%GC
*P. chrysospilos*	Tra Vinh	OP777919	1138	23.29	32.07	15.11	29.53	52.81	47.19
	Soc Trang	OP777920	1130	23.27	32.04	15.04	29.65	52.92	47.08
	Bac Lieu	OP777921	1129	23.38	32.06	14.97	29.58	52.97	47.03
	Ca Mau	OP777922	1137	23.48	32.10	14.95	29.46	52.95	47.05
*P. variabilis*	Tra Vinh	OP777923	1133	23.83	30.71	14.39	31.07	54.90	45.10
	Soc Trang	OP777924	1135	23.96	30.66	14.36	31.01	54.98	45.02
	Bac Lieu	OP777925	1136	23.77	30.63	14.52	31.07	54.84	45.16
	Ca Mau	OP777926	1131	23.87	30.50	14.41	31.21	55.08	44.92
*P. gracilis*	Tra Vinh	OP777927	1132	23.76	29.95	14.58	31.71	55.48	44.52
	Soc Trang	OP777928	1135	23.70	30.22	14.45	31.63	55.33	44.67
	Bac Lieu	OP777929	1131	23.78	30.06	14.59	31.56	55.35	44.65
	Ca Mau	OP777930	1133	23.74	30.10	14.56	31.60	55.34	44.66

Three *Periophthalmus* species were rich in C (30%–32%) but low in G (14%–15%). Similar nucleotide percentages were also recorded in studies of Cantatore et al. (1994) and Almodóvar et al. (2000). Symonová and Suh (2019) noted that the ratio of AT/GC in fish genomes was similar, and %GC was inherently low. In this study, AT/GC was about 1.2 in all sequences, and %AT was consistently greater than %GC. In the research of Çiftci et al. (2013) about the sturgeon species in the Black Sea, the %AT of the *Cytb* gene was also higher and consistently more remarkable than the %GC, similar to the report of Apostolidis et al. (1997) on *Salmo trutta* L. (Greek brown trout). The percentage of AT was about 53%–55%, suggesting that the genetic diversity of these mudskippers was moderate. The high AT was common in fish and could be found in easily hypervariable locations (Simon 1991). High AT nucleotide content is of mitochondrial DNA (Min and Hickey 2007). High AT content may result from natural selection favoring the accumulation of A and T nucleotides over G and C nucleotides in the mitochondrial genome. The exact mechanism of this AT bias is not yet fully understood (Arisuryanti et al. 2024).

### 3.3 Genetic Distance

The intraspecific and interspecific genetic differences are essential information for species classification by DNA barcode (Hebert et al. 2003; Chakraborty and Ghosh 2014). Zemlak et al. (2009) stated that fish species are considered to be the same species if the genetic distance is less than or equal to 3.5%; otherwise, they are considered different species. In the study, the conspecific genetic distances of three mudskipper species were 0% (Table 6), proving that there were no genetic variations between the individuals of each species sequenced in this study. Thus, the *Cytb* gene helped to identify three mudskipper species of the genus *Periophthalmus* accurately.

**TABLE 6:** Kimura 2‐parameter intraspecific and interspecific genetic distances of cytochrome‐*b* sequence.

Species	(1)	(2)	(3)	(4)	(5)	(6)	(7)	(8)	(9)	(10)
(1) *P. chrysospilos*	*0%*									
(2) *P. variabilis*	15%	*0%*								
(3) *P. gracilis*	18%	17%	*0%*							
(4) *P. waltoni*	16%	17%	14%	*0%*						
(5) *P. modestus*	17%	17%	17%	17%	*1%*					
(6) *P. magnuspinnatus*	16%	15%	17%	17%	17%	*0%*				
(7) *P. minutus*	16%	16%	14%	15%	15%	17%	*15%*			
(8) *P. barbarus*	17%	16%	14%	13%	16%	17%	15%	*0%*		
(9) *P. argentilineatus*	18%	17%	18%	17%	1%	17%	15%	16%	*0%*	
(10) *P. novemradiatus*	15%	1%	18%	17%	17%	15%	16%	17%	17%	*0%*

*Note:* Italic values are intraspecies distances.

The standard threshold of congeneric genetic distances in taxonomy has not yet been found (Bingpeng et al. 2018); for example, 22.2% for Indian goby species (Viswambharan et al. 2015) or 9.93% for Australian goby species (Ward et al. 2005). The K2P congeneric genetic distance in the present study fell in the 15%–18% range. Namely, the interspecific genetic distances of *P. chrysospilos* — *P. variabilis* , *P. chrysospilos* — *P. gracilis* , and *P. variabilis* — *P. gracilis* were 15%, 18%, and 17%, respectively. This result is similar to the genetic distance between *Periophthalmus* species in research and species on GenBank, such as *P. waltoni* , *P. modestus* , *P. magnuspinnatus* , *P. minutus* , *P. barbarus* , *P. argentilineatus* , and *P. novemradiatus* (13%–18%). Thus, the interspecific genetic distance of three *Periophthalmus* species in this study of about 15%–18% is reasonable. This result shows that the *Cytb* gene helped correctly identify three mudskipper species of the genus *Periophthalmus*, consistent with their morphological differences.

### 3.4 Phylogenetic Analysis

**FIGURE 2:**
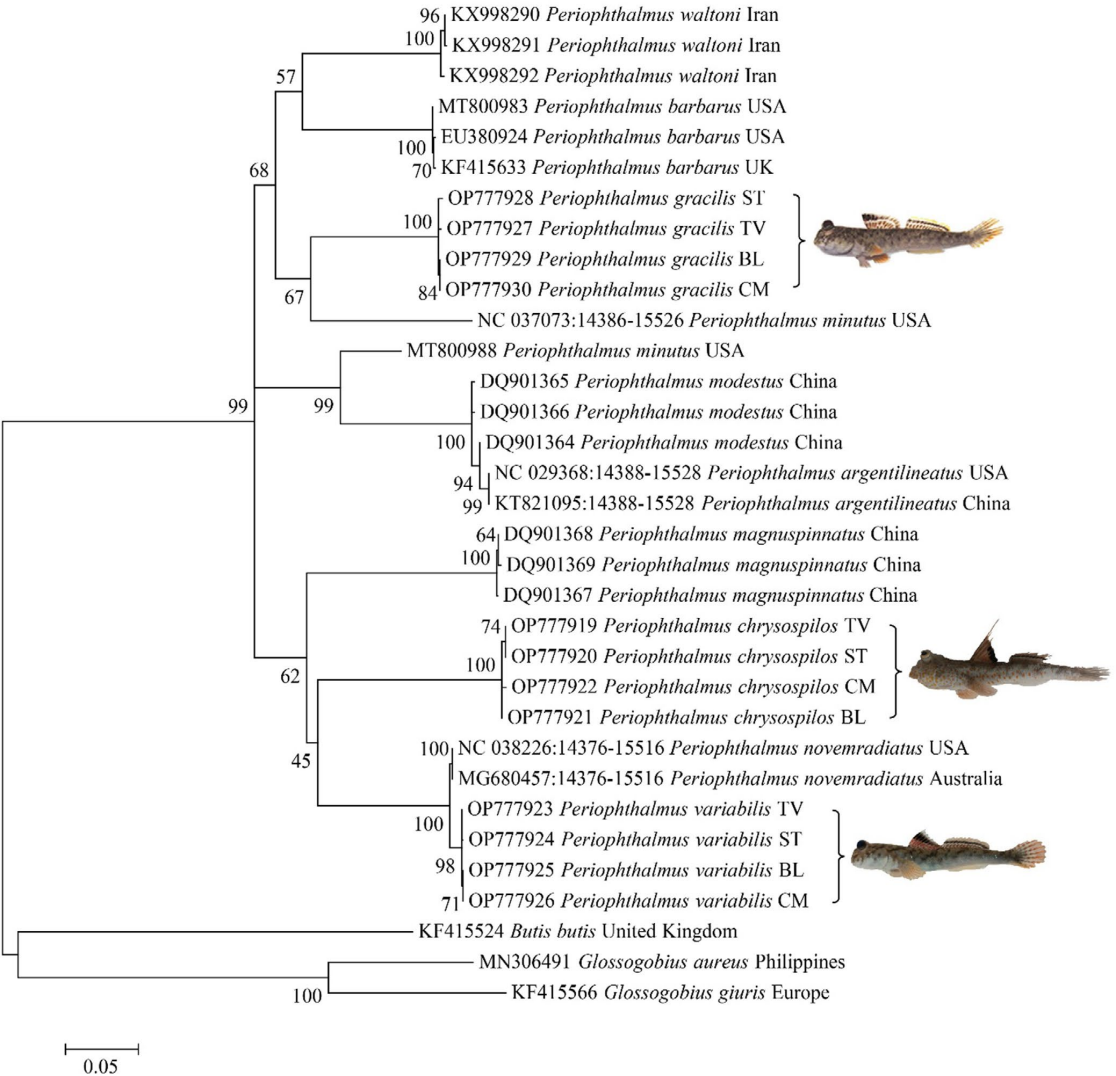
The phylogenetic tree was reconstructed using the *Cytb* gene sequence fragment by maximum likelihood (ML) based on the Hasegawa‐Kishino‐Yano +G + I mode. The values at the node represented the bootstrap values.

The phylogenetic tree in Figure 2 was separated into two main clades arising from the root of this phylogenetic tree: clade I included only one genus, *Periophthalmus*; all species of *Glossogobius* and *Butis* were grouped in clade II. The relationships between the two clades were well‐supported (99%–100% bootstrap percentage). Clade I was divided into 10 sub‐clades corresponding to three species, *P. chrysospilos* , *P. variabilis* , and *P. gracilis* in this study, and seven control species on GenBank included *P. waltoni* , *P. modestus* , *P. magnuspinnatus* , *P. minutus* , *P. barbarus* , *P. argentilineatus* , and *P. novemradiatus* . A bootstrap value of 100% supported the formation of 10 sub‐clades in the Maximum Likelihood methods, showing that these sub‐clades were sturdy and dependable. Within each sub‐clade, bootstrap values were lower than 100% (ranging from 64%–99%), possibly due to low numbers of species represented in the phylogeny, thus “missing links” reducing support.

The divergence of these sub‐clades was supported by morphological data in which *P. chrysospilos* had small orange spots, *P. variabilis* had black spots, while *P. gracilis* had horizontal bars on the body. In addition, pelvic fins in *P. variabilis* and *P. gracilis* were separated but conjugated from the disc in *P. chrysospilos*. They shared a common feature: the upper jaw had a single row of teeth. The complete morphological differences of the three species (described in Table 2) and their apparent separation in the phylogeny (Figure 2) reinforce that they are three distinct species and not due to phenotypic plasticity. This outcome was in agreement with the previous report of Jaafar and Murdy (2017) and Tran et al. (2013).

There was no geographical variation among the three mudskipper species of *Periophthalmus* genus, that is, samples from different locations belonged to the same species, suggesting a lack of geographical structure among them. Similarly, in the study of Febrianti et al. (2023), *Periophthalmus kalolo* samples collected from different locations in Indonesia belonged to the same species due to the intraspecific genetic distance of 3.5%. Another example, *P. kalolo* samples also did not show any significant genetic differentiation among populations in Indonesia (Arisuryanti et al. 2024). Interestingly, *Cytb* showed no variation along approximately 200 km of coastline in the Mekong Delta from Tra Vinh to Ca Mau. In other words, it seems that the individuals' exchange between far away populations occurs frequently enough to homogenize the genetic pool. The lack of geographical separation may be due to the small sample size. Future studies will require larger sample sizes to better understand these three species' genetic diversity patterns and geographical structure. In short, the sequences of the *Cytb* gene as a DNA barcode helped to identify the phylogeny among three species in the *Periophthalmus* genus collected from the estuaries of the Vietnamese Mekong Delta. An improved understanding of the genetic relationship of this genus is valuable in conserving genetic diversity and species management.

Mudskippers contribute significantly to the balance of the food chain in mangrove ecosystems (Budi et al. 2018). They ingest detritus and organic matter and then release nutrients into the environment (Shojaei et al. 2022). Their presence and activities in mangroves affect the ecological health and stability of these areas. Moreover, mudskippers can accumulate metal contaminants, so they are considered valuable biological indicators and monitoring agents for mangrove ecosystems (Arisuryanti et al. 2024). Accurate species identification and understanding of the genetic relationships of *Periophthalmus* mudskipper species will help conserve mudskipper species and ensure the overall ecological health of these areas.

## 4 Conclusion

All phylogenetic analysis results based on *Cytb* gene sequences showed clear differences among three species of genus *Periophthalmus* ( *P. chrysospilos* , *P. variabilis* , and *P. gracilis* ) and thus corresponded to the observed morphological differences. The intraspecific genetic distances for all three species of mudskippers were found to be 0%, suggesting that there may not be any genetic variation detected within each species. Moreover, interspecific genetic distances fluctuated between 15% and 18%, which suggested a significant genetic divergence between the three species. The study demonstrated that the combination of morphology and *Cytb* gene DNA barcode techniques was a powerful tool for identifying and understanding the genetic relationships of mudskippers. An improved understanding of the genetic relationship of these genera is valuable in conserving genetic diversity and species management.

## Author Contributions

**Quang Minh Dinh:** conceptualization (equal), formal analysis (equal), investigation (equal), methodology (equal), software (equal), supervision (equal), writing – original draft (equal), writing – review and editing (equal). **Tran Thi Huyen Lam:** formal analysis (equal), methodology (equal), software (equal), writing – original draft (equal), writing – review and editing (equal). **Gieo Hoang Phan:** investigation (equal), methodology (equal), writing – review and editing (equal). **Ton Huu Duc Nguyen:** investigation (equal), methodology (equal), visualization (equal), writing – original draft (equal), writing – review and editing (equal).

## Ethics Statement

The study was approved by the Scientific Committee of the School of Education, Can Tho. University (Animal Welfare Assessment No. BQ2020‐03/KSP).

## Conflicts of Interest

The authors declare no conflicts of interest.

## Data Availability

All data of the present study are uploaded to GenBank to get accession numbers (OP777919; OP777920; OP777921; OP777922; OP777923; OP777924; OP777925; OP777926; OP777927; OP777928; OP777929; OP777930) and presented in Table 3.
